# Insights on adaptive and innate immunity in canine leishmaniosis

**DOI:** 10.1017/S003118201600055X

**Published:** 2016-04-20

**Authors:** SHAZIA HOSEIN, DAMER P. BLAKE, LAIA SOLANO-GALLEGO

**Affiliations:** 1 Department of Pathology and Pathogen Biology, Royal Veterinary College, University of London, Hawkshead Lane, North Mymms, AL9 7TA, UK; 2 Department de Medicina i Cirurgia Animals, Facultat de Veterinària, Universitat Autònoma de Barcelona, 08193 Cerdanyola del Valles, Barcelona, Spain

**Keywords:** Canine leishmaniosis, immunology, toll-like receptors, cytokine profiles, T-cell mediated immunity

## Abstract

Canine leishmaniosis (CanL) is caused by the parasite *Leishmania infantum* and is a systemic disease, which can present with variable clinical signs, and clinicopathological abnormalities. Clinical manifestations can range from subclinical infection to very severe systemic disease. Leishmaniosis is categorized as a neglected tropical disease and the complex immune responses associated with *Leishmania* species makes therapeutic treatments and vaccine development challenging for both dogs and humans. In this review, we summarize innate and adaptive immune responses associated with *L. infantum* infection in dogs, and we discuss the problems associated with the disease as well as potential solutions and the future direction of required research to help control the parasite.

## INTRODUCTION

Leishmaniosis is a protozoan disease that is transmitted by the bite of a female phlebotomine sand-fly. Both human and canine leishmaniosis (CanL) are categorized as neglected tropical diseases, and are endemic in the Mediterranean Basin, the Middle East and in sub-tropical and tropical regions of the world (Desjeux, 2004). Canine leishmaniosis due to *Leishmania infantum* is endemic in approximately 50 countries affecting two major regions; South America and the Mediterranean region (Baneth *et al.* 2008). Prevalence within these regions is variable, largely influenced by climatic and ecological conditions, which determine the abundance of the vector (Solano-Gallego *et al.* 2009). Canine leishmaniosis spreads quickly and extensively among dog populations when conditions for transmission become favourable; that is when there is a high sand-fly vector and canine-host density (Baneth *et al.* 2008; Espejo *et al.* 2015). Precise prevalence rates are difficult to compare as over the years different methods have been used to detect infection. Early epidemiological studies were based on direct parasitological and serological tests, whereas recent observations include molecular or cellular immunological methods (Baneth *et al.* 2008). However, there is a positive relationship between the prevalence of disease in human and canine populations (Marty *et al.* 1992; Cunha *et al.* 1995). A high prevalence of CanL in the Mediterranean region is present, while human leishmaniosis is hypoendemic (Farrell, 2002). Increases in human *Leishmania* infections in this region have been linked to an increase in human immunodeficiency virus cases and co-infection (Abdalmaula *et al.* 2013), but there is growing evidence that disease is also diagnosed in immunocompetent adults (Gramiccia *et al.* 2013; Horrillo *et al.* 2015).

*Leishmania* has a digenetic lifecycle. *Leishmania* metacyclic promastigotes are injected under the skin of the vertebrate host by the phlebotomine (sand fly) vector. This form of the parasite is flagellated and they are rapidly engulfed by phagocytic cells such as neutrophils, dendritic cells and macrophages, which are either resident or recruited to the bite site (Kaye and Scott, 2011). It is believed that the parasites use neutrophils as a ‘safe hideaway’, but establish prolific infections in macrophages where the flagellated promastigotes differentiate into amastigotes (Faria *et al.* 2012). These amastigotes then replicate within the parasitophorous vacuole. Amastigotes multiply by binary division, causing the host cell to enlarge and eventually rupture, allowing the released amastigotes to infect other mononuclear phagocytic cells. Amastigotes circulating in the blood stream are then available to be ingested by a sand fly when it takes a blood meal and the cycle repeats itself (Wakelin, 1996).

The immune responses to *L. infantum* infection in dogs are very variable and can play a key role in controlling the infection. There is a large body of work that focuses on T-cell mediated immunity but there is limited data on the importance of innate immune responses in CanL. The aim of this paper was to bring together current knowledge of the adaptive and innate immune responses associated with *L. infantum* infection in dogs.

## CANL

Dogs appear to be the main reservoir host for *L. infantum* infection, however, in many regions other canids such as jackals, foxes, wolves and other mammals, such as hares or black rats, have been implicated as wild reservoirs (Millan *et al.* 2014). It has been suggested that domestic cats should receive greater attention as a public health risk, since feline leishmaniosis has been reported in many countries where canine and human disease is endemic (Martin-Sanchez *et al.* 2007; Maia *et al.* 2008).

Not all dogs exposed to *L. infantum* develop clinical manifestations and subclinical infections are more frequent than clinical disease. Therefore, *L. infantum* in dogs can manifest as chronic subclinical infection, self-limiting disease or severe illness (Solano-Gallego *et al.* 2009). The outcome of canine *L. infantum* infection is largely influenced by the development of the cell-mediated immune (CMI) response. T lymphocytes play a crucial role in immunity to leishmaniosis by influencing cytokine production and subsequently interacting with infected macrophages (Rodriguez-Cortes *et al.* 2007*a*).

In dogs, which become sick, clinical features and the incubation period can vary extensively (Solano-Gallego *et al.* 2011). A clinical staging system based on clinico-pathological abnormalities, clinical signs and serological status has been proposed in an attempt to cover the wide spectrum of clinical manifestations found in CanL (Solano-Gallego *et al.* 2009). Based on this system, the correct treatment can be determined and a realistic prognosis be ascertained. The most common clinical signs include lymphadenomegaly, cutaneous lesions, weight loss, anorexia, lethargy and ocular lesions (Solano-Gallego *et al.* 2011). Epistaxis is likely to be the result of several pathogenic factors such as nasal-mucosal ulceration and thrombocytopathy (Petanides *et al.* 2008). An increased number and size of lymphoid follicles results in lymphadenomegaly, facilitating palpation of superficial lymph nodes as a diagnostic measure (Giunchetti *et al.* 2008). Splenomegaly might also be noted, but is often mild and difficult to detect. This results from a disorganization of normal lymphoid tissue and loss of normal spleen leukocyte diversity (Santana *et al.* 2008). Hepatomegaly and liver disease including macrophagic and lymphoplasmacytic inflammation and collagen deposition has also been reported in cases of CanL (Rallis *et al.* 2005; Melo *et al.* 2009), although the principal cause of death attributed to CanL is severe renal damage (Solano-Gallego *et al.* 2009), with glomerulonephritis resulting from immune complex deposition associated with an intense humoral response and high parasite loads (Costa *et al.* 2003).

## IMMUNE VARIATION AS A RISK FACTOR ASSOCIATED WITH CANL

The precise immune mechanisms responsible for resistance or susceptibility to infection and subsequent disease are not known for CanL. Nonetheless, it appears that factors such as breed, immunosuppression, concomitant disease and nutritional status can influence the outcome of infection (Miro *et al.* 2008). Similarly, the presence of ongoing co-infections with pathogens such as *Ehrlichia canis* or Filarias or previous infections appear to be associated with more clinical signs and clinicopathological abnormalities and disease severity (Mekuzas *et al.* 2009; Cortese *et al.* 2011; Tabar *et al.* 2013). Other factors that might determine the outcome of infection includes the virulence of *L. infantum* parasites (Martin-Martin *et al.* 2015), inoculum of parasites, sand-fly transmission or other routes of transmission. For example, sand fly salivary proteins can induce a strong cellular immune response in dogs (Collin *et al.* 2009). One study demonstrated that dogs developed a strong immune response to two salivary proteins, namely LJL143 and LJM17 from the natural vector sand fly *Lutzomyia longipalpis*. Blood collected from these immunized dogs was found to contain cells that were producing interferon-gamma (IFN-*γ*) and subsequently resulted in the recruitment of immune cells at the inoculation site, which were found to have an adverse effect on *Leishmania* parasites (Collin *et al.* 2009).

All breeds of dog are potentially susceptible to *L. infantum* with factors such as age, gender and proportion of time spent outdoors (especially overnight) found to influence the likelihood and outcome of infection (Franca-Silva *et al.* 2003; Zivicnjak *et al.* 2005; Miranda *et al.* 2008; Galvez *et al.* 2010). However, it has also been established that mongrels and autochthonous breeds from endemic areas develop various degrees of resistance to disease (Solano-Gallego *et al.* 2000). An example of such a breed is the Ibizan hound from the island of Mallorca (Spain). The breed develops very effective cellular immune responses and rarely presents with clinical disease (Solano-Gallego *et al.* 2000). Amongst pedigree dogs, studies have found that breeds such as Rottweilers, Boxers and German Shepherds are more susceptible to disease than other breeds like Yorkshire Terriers and Poodles (Ciaramella *et al.* 1997; Franca-Silva *et al.* 2003; Miranda *et al.* 2008). It has also been reported that Foxhounds may have a genetic susceptibility to CanL (Duprey *et al.* 2006). One component of such genetic variation is the nature of the immune response against *L. infantum* infection (Baneth *et al.* 2008).

Dog leukocyte antigen (DLA) is part of the major histocompatibility complex (MHC) in dogs. DLA has been found to be the main genetic component associated with canine diabetes. In prone breeds (including Samoyed, Cairn Terrier and Tibetan Terrier), the common alleles/haplotypes are DLA-DBR1*009, DQA1*001 and DQB1*008 (Catchpole *et al.* 2008). Quinnell *et al.* reported a study, which investigated the relationship between DLA class II alleles and the course of *L. infantum* infection in a group of naturally infected Brazilian dogs. The study revealed a significant association between the presence of the allele DLA-DRB1*01502 and susceptibility to CanL (Quinnell *et al.* 2003*b*). The dogs presenting this allele also had significantly higher anti*-Leishmania* IgG levels and were far more likely to be parasite positive by polymerase chain reaction (PCR). High IgG levels have been associated with susceptibility (Quinnell *et al.* 2003*b*). Polymorphisms and mutations of the solute carrier family 11, member a1 gene (Slc11a1) have also been shown to exert a genetic influence. The gene, also known as natural resistance-associated macrophage protein 1 (NRAMP1), encodes an ion transporter protein, which is involved in the control of intraphagosomal replication of parasites and in macrophage activation (Blackwell *et al.* 2001). The haplotype TAG-8-141 has been associated with Boxer breed predisposition to CanL (Sanchez-Robert *et al.* 2005). It is believed that numerous loci are accountable for the progression of disease as a result of *L. infantum* infection in dogs (Quilez *et al.* 2012). Quilez *et al.*  performed a genome-wide scan using a dataset of 115 healthy infected and 104 affected Boxer dogs and identified over 126 000 single nucleotide polymorphisms (SNPs) distributed across the genome. Analysis of these SNPs allowed the phenotype to be predicted with an accuracy of ~0·29 in new samples, providing evidence of a significant genetic component to CanL within the breed (Quilez *et al.* 2012). A recent study using a genome-wide scan in 20 mixed–breed dogs and 28 controls identified two candidate loci in chromosomes 1 and 2. Markers discovered were related to notch signaling (critical for macrophage activity and cluster of differentiation 4 (CD4) T cell differentiation) and IL-2, IL-15 and IFN-*γ* expression (Utsunomiya *et al.* 2015). In addition, polymorphisms in canine toll-like receptors (TLRs) were characterized following sequencing after enrichment of exonic regions (Cusco *et al.* 2014). Seven different breeds were studied and DNA from a total of 335 dogs and 100 wolves were included in the pools. The results revealed that TLR5 was the most polymorphic amongst all canine TLRs (Cusco *et al.* 2014).

## IMMUNE RESPONSES ASSOCIATED WITH CANL

Immune-mediated mechanisms are responsible for many of the pathological findings in CanL. Circulating immune complexes and anti-nuclear antibodies have been detected in dogs with CanL (Lucena and Ginel, 1998; Margarito *et al.* 1998; Smith *et al.* 2004). Vasculitis induced by immune complexes activates the complement cascade (Torrent *et al.* 2005). This is an important pathological mechanism, which is responsible for tissue necrosis and for some of dermal, visceral ocular and renal lesions commonly found in CanL (Vamvakidis *et al.* 2000; Torrent *et al.* 2005; Baneth *et al.* 2008). The severity of disease is correlated with high antibody levels and increasing parasite load (Farrell, 2002). In addition, a study has revealed that the number of apoptotic cells within the inflammatory infiltrate is directly related to the parasitic load and thus the severity of the clinical picture in infected dogs (Vercosa *et al.* 2012).

*Leishmania* is renowned for its ‘silent’ establishment of disease, illustrated by little or no change in immune response despite parasite proliferation and dissemination. A silent phase was reported by Belkaid *et al*. in mice infected with *L. major* over a period of 4–5 weeks before the development of lesions (Belkaid *et al.* 2000). This silent establishment was also reported later in dogs (Santos-Gomes *et al.* 2002). A low proportion of experimentally infected dogs expressed specific cytokines in the first 8 months of infection coupled with parasite dissemination, but without clinical signs of disease (Santos-Gomes *et al.* 2002). The authors suggested that this was the parasite's ‘silent establishment’ by avoiding adverse host-cell mediated immunological reactions (Santos-Gomes *et al.* 2002).

The following sections of this paper will be about the immune responses associated with CanL that can be further broken down into adaptive and innate immune responses. We will report the adaptive immune responses first, as these responses have been covered in greater detail over a longer period of time compared with innate responses.

## ADAPTIVE IMMUNITY

In early studies a clearly defined Th1/Th2 (pro-inflammatory/anti-inflammatory) dichotomy was observed in murine leishmaniosis (Sadick *et al.* 1986; Bretscher *et al.* 1992; Menon and Bretscher, 1998), however it has since become apparent that the immune responses associated with both human and CanL are far more complex. Nonetheless, it is clear that in both human and experimental animal models of the disease, cytokines play a very important role in influencing the nature of the host immune response (Cummings *et al.* 2010). The ability of the host to control *L. infantum* infection requires the generation of CMI responses, which activate host macrophages, eliminating intracellular parasites. In both human and experimental models of cutaneous leishmaniosis (CL), control of the infection is mediated by the early induction of an IL-12 driven Th1 immune response along with the production of IFN-*γ* by CD4^+^ T cells (Tripathi *et al.* 2007). Conversely, susceptibility to infection and disease progression in CL is driven predominantly by the induction of a non-protective IL-4 Th-2-type response and the production of Th-2 associated cytokines such as IL-4, IL-10, IL-13 and transforming growth factor (TGF)-*β* (Alexander and Bryson, 2005; Tripathi *et al.* 2007). In experimental models of murine CL, IL-4 has been shown to play a crucial role in mediating susceptibility to *Leishmania* infection by the down regulation of protective Th-1 associated cytokines IL-12 and IFN-*γ*, therefore inhibiting nitric oxide (NO) production and eventual parasite killing by macrophages (Alexander *et al.* 1999). However, further studies later demonstrated that the IL-4 mediated exacerbation of CL was dependent upon the particular strain of *Leishmania* (Kropf *et al.* 2003).

Unlike mice, immune responses seen in dogs more closely resemble those seen in human infections. Many early CanL studies described protective cell mediated immune responses that were associated with the activation of Th1 cells, producing IFN-*γ*, IL-2 and TNF-*α*, and active disease characterized by mixed Th1/Th2 responses (Carrillo and Moreno, 2009). However, many of these investigations were performed on peripheral blood samples and further studies indicated that the immune responses to the parasites were in-fact organ specific (Reis *et al.* 2009). Th1, Th2 or mixed Th1/Th2 immune responses were subsequently observed in different organs of CanL dogs. These correlated with the absence or presence of clinical signs and parasite load data (Strauss-Ayali *et al.* 2007; Travi *et al.* 2009; Boggiatto *et al.* 2010). These findings illustrated that the phenotypic cell profiles and cytokines involved in immune responses, in compartments where parasites are known to replicate, have variable effects on local parasite control, highlighting the complexity of cellular immune responses in *L. infantum* infection (Maia and Campino, 2012). Indeed, cytokine profiles in different tissues vary greatly. Table 1 shows examples of studies evaluating cytokine changes in several compartments that have been evaluated in natural and experimental canine *L. infantum* infection to date. It is important to highlight the differences between studies on clinical classification of dogs, type of infection, technique employed for measurement of cytokines and geographical location as well as the small sample size of dogs evaluated in the majority of these studies.

**Table 1.** Examples of studies evaluating cytokine changes in several compartments in natural and experimental canine *L. infantum* infection

Cytokines/tissues evaluated	Technique employed for cytokine detection	Clinical status classification and number of dogs studied	Geographical location	Main findings	Reference
IL-10, IL-17A, TNF-*α*, IFN-*γ*/liver, spleen	RT–PCR	AD (*n* = 11)	Brazil	↑Parasites and ↓leucocyte infiltration in liver in SD dogs compared with AD dogs.	(Nascimento *et al.* 2015)^a^
		SD (*n* = 10)			
				Disease progression characterized by ↓ of Th1 cytokines, iNOS and IL-10 in the liver and spleen.	
				IL17A gene transcription positively correlated with iNOS and IFN-*γ*	
IL-6, IL-10, IL-12,TGF-*β*, IFN-*γ*, TNF-*α*/spleen	ELISA, RT–PCR	Seropositive dogs: Low parasite (*n* = 46)	Brazil	↓Cytokine transcripts in the high parasite load group	(Cavalcanti *et al.* 2015)^a^
		High parasite (*n* = 42)			
				Negative correlation was found TNF-*α*, IFN-*γ*, IL-12, IL-6; and: IL-10 and TGF-*β* and parasite load	
IL-10, IFN-*γ*/serum, spleen	ELISA, RT–PCR	Seropositive dogs (*n* = 44) based on parasite load in the spleen were divided into Group 1: healthy or mild disease	Brazil	↑ IFN-*γ* and TNF-*α* was associated with reduced *Leishmania* burden, whereas ↑ IL-10 was associated with higher *Leishmania* load	(Nascimento *et al.* 2013)^a^
		Groups 2–4: sick dogs			
IL-4, IL-10, TNF-*α*/spleen, liver	ELISA, RT–PCR	Non-infected (*n* = 12)	Brazil	↑ IL-4, IL-10 and TNF-*α* in infected dogs	(Michelin *et al.* 2011)^a^
		AD(*n* = 20) SD (*n* = 20)		Splenic TNF-*α* was correlated with parasite load	
IL-4, IL-10, IL-12, IFN-*γ*, TNF-*α*/spleen	RT–PCR	Non-infected (*n* = 7)	Brazil	↑ IL-12 production in infected dogs compared with non-infected controls.	(Lage *et al.* 2007)^a^
		AD (*n* = 8)			
		OD (*n* = 10)			
		SD (*n* = 12)			
				IL-10 was positively correlated with disease progression and parasitism.	
				Positive correlation between parasitism and IFN-*γ*	
IL-10, TGF-*β*1, IFN-*γ*/spleen, liver	ELISA	AD (*n* = 15)	Brazil	↑TGF-*β*1 in AD in both liver and spleen.	(Correa *et al.* 2007)^a^
		SD (*n* = 15)			
				↑IL-10 in both liver and spleen of both groups.	
				↑IFN-*γ* in liver of AD and spleen of SD	
IL-17, IL-22/lymph node, liver, spleen, skin	RT–PCR	Non-infected healthy (*n* = 10)	Spain and UK	IL-22 transcription down regulation with disease progression in the liver, spleen and lymph nodes	(Hosein *et al.* 2015)^b^
		Sub-clinical (*n* = 24)			
		Sick (*n* = 7)			
				No changes of IL-17 with disease progression	
IFN-*γ*, TNF-*α*, IL-4, IL-5, IL-10, IL-18, IL-12p40, TGF-*β*/spleen	RT–PCR	Non-infected (*n* = 2) Experimentally infected (*n* = 7)	Israel	Splenic IFN-*γ* increased most significantly of all cytokines following infection.	(Strauss-Ayali *et al.* 2007)^a^^,^^b^
		Naturally infected (*n* = 10)		No changes in IL-18, IL-12, TNF-*α*, IL-10, TGF-*β* expression were noted during infection	

IFN-*γ*, TNF-*α*, TGF-*β*, IL-10/lymph node	RT–PCR	Non-infected (*n* = 6)	Brazil	↑ IFN-*γ* and TNF-*α* along with low parasite burden in AD.	(Alves *et al.* 2009)^a^
		AD (*n* = 6)			
		SD (*n* = 6)			
				↑ IL-10 and TGF-*β* with disease progression and high parasite load	
IL-2, IL-4, IL-12, IFN-*γ*/blood, lymph node, bone marrow	IFA, ELISA	Non-infected Healthy (*n* = 11)	Portugal and Brazil	AD: ↑IL-4 in blood leucocytes and IL-12 in lymph nodes.	(Barbosa *et al.* 2011)^a^
		AD (*n* = 13)			
				SD: ↑IL-2 and IFN-*γ* in lymph nodes and bone marrow appeared non-responsive	
		SD (*n* = 29)			
		Treated (*n* = 11)			
IL-10, IFN-*γ*/*L. infantum* stimulated PBMC	ELISA	Non-infected (*n* = 2)	USA	Disease progression correlates with ↓ IFN-*γ* and ↑ IL-10 production	(Boggiatto *et al.* 2010)^a^
	RT–PCR	Infected resistant (*n* = 4)			
		Infected susceptible (*n* = 3)			
		Clinical (*n* = 4)			
IL-6, TNF-*α*/blood	ELISA	Healthy (*n* = 8)	Brazil	↑IL-6 was found in infected dogs	(de Lima *et al.* 2007)^a^
		Infected (*n* = 16)			
				No differences in TNF-*α* production were reported between groups	
IL-4, IL-10, IL-13, IL-18/blood, bone marrow	Semi-quantitative PCR	Non-infected controls (*n* = 5)	Brazil	↑ IFN-*γ* in infected dogs in both organs	(Quinnell *et al.* 2001)^a^
		Infected (*n* = 27)		Infected: ↑ IL-4 in bone marrow	
IL-2, IL-4, IL-10, IL-18, TNF-*α*, IFN-*γ*/unstimulated blood, *ex vivo* stimulated blood	RT–PCR	Non-infected (*n* = 5)	Spain	Cytokine levels were similar in both groups of dogs in unstimulated blood.	(Chamizo *et al.* 2005)^b^
		AD (*n* = 7)			
				Stimulation with soluble leishmanial antigen promoted ↑ IL-2 and IFN-*γ*	
IFN-*γ*, IL-2, IL-12p40, IL-6, IL-10/stimulated blood	RT–PCR	Non-infected controls (*n* = 2)	Portugal	In the first 8 months, low number of dogs expressing specific cytokines, indicative of ‘silent establishment’ of the parasite	(Santos-Gomes *et al.* 2002)^b^
		Infected (*n* = 13)			
IL-4, IL-5, IL-10, IL-12, IL-3,TGF-*β*1, IFN-*γ*, TNF-*α*/skin	RT–PCR	Controls (*n* = 16)	Brazil	Mixed cytokine profile observed during active disease.	(Menezes-Souza *et al.* 2011)^a^
		AD (*n* = 10)			
		OD (*n* = 10)			
		SD (*n* = 15)		An impairment in the expression of both TNF-*α* and IFN-*γ* correlated with the morbidity of disease	
IL-4, IL-13, TNF-*α*, IFN-*γ*/skin	RT–PCR	Healthy non-infected controls (*n* = 10)	Italy	↑ IL-4, TNF-*α* and IFN-*γ* in sick dogs	(Brachelente *et al.* 2005)^a^
		Infected sick dogs (*n* = 10)			
IL-4, IFN-*γ*/unstimulated blood	Immunofluorescence Flow cytometry	Controls	Italy	The percentage of Th1 Tcells producing IFN-*γ* in LD animals was significantly higher compared with controls, irrespective of the grouping criteria used	(Cortese *et al.* 2013)^a^
		LD-S – (*n* = 22)			
		LD-A – (*n* = 23)			

AD, asymptomatic; SD, symptomatic; OD, oligosymptomatic; LD, *Leishmania* infected dogs; LD-S, clinical signs and clinical pathological abnormalities; LD-A, no clinical signs or clinical pathological abnormalities; iNOS, inducible nitric oxide; IL, interleukin; IFA, immunofluorescence; IFN, interferon; TGF, transcription growth factor; TNF, tumour necrosis factor; ELISA, enzyme linked immunosorbent assay; RT–PCR, reverse transcriptase and real time polymerase chain reaction; PBMC, peripheral blood mononuclear cells.

a Natural infection.

b Experimental infection.

## ORGAN SPECIFIC IMMUNE RESPONSES

### Lymph node

In popliteal lymph nodes, a balance was observed between the percentage of IL-10 and TNF-*α* expression, which was possibly attributed to an absence of clinical signs and low parasite loads (Maia and Campino, 2012). This was similar to earlier findings where lymph nodes from sub-clinical dogs had a high expression of pro-inflammatory cytokines, which was correlated with a low number of parasites. The authors suggested a role for IL-10 and TGF-*β* in disease progression (Alves *et al.* 2009). In a study by Barbosa *et al.* they reported that asymptomatic dogs showed high expression of genes encoding IL-2 and IL-12 in lymph nodes (Barbosa *et al.* 2011). Hosein *et al.* reported down regulation of IL-22 with disease progression in an experimental CanL model (Hosein *et al.* 2015). Giunchetti *et al*. reported that lymph node CD8^+^ T cells might be involved in a distinct activation status during CanL which was most likely associated with immunomodulatory or suppressor cell activity (Giunchetti *et al.* 2008). Alexandre-Pires *et al*. demonstrated that CD8^+^ T subpopulations in lymph nodes from treated dogs were significantly lower than in asymptomatic non-treated dogs. In addition, they reported that the CD4^+^ T cell subset in lymph nodes of both treated and asymptomatic dogs was significantly higher than that in control, non-infected dogs (Alexandre-Pires *et al.* 2010). These findings possibly indicate that an increase in CD8^+^ T cells is associated with parasite persistence, whereas CD4^+^ subpopulation expansion favours control of the parasite (Alexandre-Pires *et al.* 2010; Maia and Campino, 2012).

### Liver

In the liver of CanL dogs, production of IFN-*γ*, IL-10 and TGF-*β* was reported to be high in naturally infected dogs with no clinical signs of the disease (Correa *et al.* 2007). Maia *et al.* reported similar findings a few years later. Experimentally infected dogs expressed these cytokines in addition to inducible NO synthase (iNOS). However, IL-4 and TNF-*α* were not expressed (Maia and Campino, 2012). It was suggested that the absence of TNF-*α* in these experimentally infected dogs might have been associated with a high level of parasitism (Maia *et al.* 2010; Maia and Campino, 2012). Hosein *et al.* also reported a significant down regulation of IL-22 transcription in both liver and spleen samples when non-infected controls were compared with *L. infantum* infected dogs (Hosein *et al.* 2015). Nascimento *et al.* investigated chemokine and cytokine receptor expression in dogs naturally infected with *L. infantum* (Nascimento *et al.* 2013). In the liver of symptomatic dogs, a significant down regulation was noted for expression of beta chemokine receptors (CCR), CCR3, CCR4, CCR5, CCR6 and CCR8 when compared with expression levels in asymptomatic dogs. These chemokines were also downregulated when symptomatic dogs were compared with healthy non-infected controls (Nascimento *et al.* 2013). The authors concluded that the suppression of these receptors seen in symptomatic dogs was possibly due to a reduced migration of immune cells, which would normally assist with parasite clearance and granuloma formation (Nascimento *et al.* 2013). Recently, the same authors documented that the progression of this disease was characterized not only by the down regulation of T helper (Th) 1-related cytokines, such as IFN-*γ* and TNF-*α*, but also by the down regulation of genes encoding interleukin (IL)-17A, inducible NO synthase (iNOS) and IL-10 in the spleen and liver in symptomatic dogs compared with asymptomatic dogs (Nascimento *et al.* 2015).

### Spleen

In CanL, there is a strong association with splenic architecture disruption, often characterized by the disorganization of normal lymphoid tissue, a loss of normal spleen leukocyte diversity and eventual atrophy of the lymphoid tissue (Sanchez *et al.* 2004). It was reported that in natural infection there was a positive correlation between the expression of IL-10 with increasing parasite load and disease progression (Lage *et al.* 2007). Conversely, it has also been reported that no changes in IL-10 expression were seen in experimentally infected dogs irrespective of parasite load (Strauss-Ayali *et al.* 2007). In addition, an association between high IFN-*γ* expression and chemokine expression, and splenic parasitism with disease progression was reported (Correa *et al.* 2007). Cavalcanti *et al*. recently reported that in naturally infected dogs, parasite load induced splenic architecture breakage, which resulted in an impairment of both pro-inflammatory (IFN-*γ*, IL-2 and IL-6) and anti-inflammatory cytokines (IL-10 and TGF-*β*) (Cavalcanti *et al.* 2015). Michelin *et al*. measured TNF-*α*, IL-4 and IL-10 in the liver and spleen of dogs naturally infected with *L. infantum* and reported that all cytokines studied were higher in the spleens of symptomatic dogs compared with asymptomatic or non-infected controls (Michelin *et al.* 2011).

Hosein *et al*. reported an involvement of IL-22 in the spleen of experimentally infected dogs. In both early and late stages of infection (6 and 15 months post infection), IL-22 transcription was significantly lower when compared with non-infected healthy control dogs (Hosein *et al.* 2015). Information about the involvement of IL-22 in CanL is scarce but in human studies, it has been described that lower levels of IL-22 in human visceral leishmaniosis (VL) is associated with disease establishment and it has been suggested that IL-22 plays a complementary role to Th1 cytokines in the protection against VL (Pitta *et al.* 2009).

Nascimento *et al.* also reported changing chemokine and chemokine receptor profiles in the spleens of dogs with varying clinical forms of CanL (Nascimento *et al.* 2013). The authors looked at chemokines from the CC-family chemokine ligand family (CCL), chemokine motif ligand (CXCL), CXC chemokine receptors (CXC) and beta chemokine receptors (CCR). A reduction in expression levels of CCL1, CCL3, CCL17, CCL20, CCL26, CXCL9, CCR3, CCR4, CCR6 and CCR8 was reported in the spleens of both symptomatic and asymptomatic dogs when compared with non-infected controls. The mRNA levels of CCL2, CCL5 and CXCL10 were however increased in the spleens of symptomatic dogs compared with the levels in the spleens of asymptomatic animals (Nascimento *et al.* 2013).

### Bone marrow

The progression of *Leishmania* infection has been linked with macrophagic inflammation in bone marrow, accompanied by an increased percentage of plasma cells and lymphocytes as well as erythroid hypoplasia (Maia and Campino, 2008). Quinnell *et al.* observed an increased accumulation of TNF-*α* and IFN-*γ* in the bone marrow of naturally infected dogs (Quinnell *et al.* 2001), and these findings were also similar to those of Manzillo *et al.* who speculated that erythroid hypoplasia were attributable to bone marrow macrophages producing high levels of these cytokines (Manzillo *et al.* 2006*b*). Foglia Manzillo *et al.* evaluated bone marrow aspirates from 15 naturally infected dogs with and without clinical signs of disease (Manzillo *et al.* 2006*b*). They reported that the most common pathological features within the bone marrow of these dogs were megakaryocytic dysplasia, which was noted in 10 of the 15 dogs, and erythrophagocytic dysplasia in eight dogs. These features were also thought to be linked to the increased number of macrophages found to be producing high levels of IFN-*γ* and TNF-*α* (Manzillo *et al.* 2006*b*). Barbosa *et al.* reported significantly higher levels of IL-12 mRNA in the bone marrow of dogs following treatment with allopurinol and meglumine antimoniate compared with healthy, asymptomatic and symptomatic dogs (Barbosa *et al.* 2011).

### Skin

The skin is crucial for parasite transmission and skin parasite burdens seem to be the best marker for transmission potential (Courtenay *et al.* 2014). Guarga *et al.* determined that even seropositive subclinical dogs were infectious to sand-flies through xenodiagnosis (Guarga *et al.* 2000). Madeira *et al*. looked at skin as a potential parasitological diagnostic tool for CanL and obtained skin samples from the ear and scapular regions of sero-reactive dogs in the Belo Horizonte region of Brazil (Madeira *et al.* 2009). In 61% of the animals tested, isolation of *L. infantum* was possible from ear and scapular skin. The results from the study demonstrated that intact skin could be a possible detection site for *Leishmania* parasites in sero-positive dogs (Madeira *et al.* 2009). While the skin is essential for natural transmission via sand-flies, there is limited data on the cytokine profiles in this tissue in canine *L. infantum* infection. Papadogiannakis and Koutinas recently reported a mixed Th1/Th2 cytokine profile in the dermis of dogs naturally infected with *L. infantum*. Dogs with clinical signs of CanL were found to have an overproduction of IL-4, IL-13 and TNF-*α* in the dermis (Papadogiannakis and Koutinas, 2015). Menezes-Souza *et al*. also reported a mixed cytokine profile in the skin of naturally infected dogs (Menezes-Souza *et al.* 2011). Subclinical dogs were found to express high levels of IL-13, TNF-*α* and IFN-*γ*, and trans-acting T-cell specific transcription factor GATA-3 (Menezes-Souza *et al.* 2011). An earlier study by Brachelente *et al*. looking at cutaneous lesions in naturally infected dogs revealed that dogs with high parasite burden had significantly high IL-4 expression (Brachelente *et al.* 2005). Most recently, Hosein *et al.* reported a marginal significant increase in the transcription factor forkhead box P3 (FoxP3) in both subclinical and sick dogs compared with non-infected controls (Hosein *et al.* 2015). A study by Menezes-Souza *et al.* investigated the chemokines in the skin of dogs with CanL (Menezes-Souza *et al.* 2012). It was reported that chemokine mRNA expression demonstrated enhanced parasite density and was positively correlated with the expression of CCL2, CCL4, CCL5, CCL21, and CXCL8. In contrast, there was a negative correlation between parasite density and CCL24 expression (Menezes-Souza *et al.* 2012).

### Other tissues

Other groups have investigated the cytokine profiles present in other tissues such as the brain, jejunum and colon that were deemed less commonly associated with *L. infantum* infection in dogs (Figueiredo *et al.* 2013; Melo *et al.* 2013). Figueiredo *et al*. reported higher levels of IL-10 and TNF-*α* in the jejunum than the colon of infected animals, whereas IL-4 was higher in the colon than the jejunum of infected animals (Figueiredo *et al.* 2013). In the brains of CanL dogs, Melo *et al.* reported that IL-10, TGF-*β* and IL-12p40 were downregulated, while IL-1-*β*, IFN-*γ* and TNF-*α* were upregulated, however expression levels did not correlate with parasite load (Melo *et al.* 2013). The authors suggested that the differences seen in the brain were due to the host's immune response, regardless of the phase of the disease (Melo *et al.* 2013).

## T CELL MEDIATED IMMUNITY INCLUDING REGULATORY T LYMPHOCYTES

Clinical CanL has been associated with immunological changes involving T cells (Barbieri, 2006). Such changes can be measured as the absence of delayed type hypersensitivity to *Leishmania* antigens (Pinelli *et al.* 1994; Cardoso *et al.* 1998), IL-2 production and TNF-*α* by peripheral blood mononuclear cells *in vitro* (Pinelli *et al.* 1995; Santos-Gomes *et al.* 2002), and IFN-*γ* absence and decreased T-cell numbers in the peripheral blood. There is also evidence that the absence or decreased T-cell mediated immunity is due to T-cell exhaustion (Esch *et al.* 2013). It was demonstrated that exhaustion was associated with a significant increase in the population of T cells with surface expression of programmed death 1 (PD-1). Blocking PD-1 encouraged the return of CD4^+^ and CD8^+^ T cell function and also resulted in a marked increase in the production of reactive oxygen species in co-cultured monocyte derived phagocytes (Esch *et al.* 2013). Infected macrophages were also lysed by CD8^+^ cytotoxic T cells in a histocompatibility complex-restricted process that can be supressed in sick dogs with high parasite loads (Pinelli *et al.* 1994; De Luna *et al.* 1999). In sub-clinical infection, the opposite can be seen; low parasitism and the increased prevalence of CD8^+^ T lymphocytes (Reis *et al.* 2006*a*). The vast majority of *Leishmania* infected dogs present a CMI response, which is exemplified by strong *in vitro* lymphocyte proliferation and positive skin reaction to the intradermal leishmanin injection (Montenegro test)(Cardoso *et al.* 1998; Maia and Campino, 2008). These responses are eventually blunted with disease progression in addition to decreased intracellular killing of amastigotes by neutrophils and macrophages (Brandonisio *et al.* 1996; Baneth *et al.* 2008).

In sick dogs, the depletion of T lymphocytes in lymphoid organs becomes falsely compensated B-cell proliferation and activity (Koutinas and Koutinas, 2014). This activity, along with the activity of plasma cells, histiocytes and macrophages, potentially explains the generalized lymphadenomegaly, splenomegaly and hyperglobulinemia, which have become clinical hallmarks of this disease (Baneth *et al.* 2008; Koutinas and Koutinas, 2014). CanL associated hyperglobulinemia is detrimental via the generation of auto-antibodies, anti-histone antibodies and/or the circulating immune complexes, which are generated in abundance (Lopez *et al.* 1996; Cortese *et al.* 2009). Renal complications are often a result of CanL and although there have been high incidences of infection-mediated glomerulonephritis, information about the pathogenesis of CanL associated disease is scarce. Esch *et al*. recently reported that asymptomatic and symptomatic dogs had increased glomerular nucleotide-binding domain leucine-rich repeat-containing-like receptor family, pyrin domain containing 3 and autophagosome-associated microtubule-associated protein 1 light chain 3, which was associated with lesion severity of the glomeruli (Esch *et al.* 2015). Glomerulonephritis was initiated by complement deposition and IgG, and the overall findings from the study suggest possible roles for inflammasome complexes as a cause of glomerular damage during *L. infantum* infection (Esch *et al.* 2015).

Regulatory T cells (Tregs), have an important role in suppression of host immunity in murine (Peters and Sacks, 2006; Rodrigues *et al.* 2009) and human leishmaniosis (Katara *et al.* 2011; Rai *et al.* 2012). Tregs have also been found to play a role in CanL. Silva *et al*. evaluated IL-10 and TGF-*β* production by Treg cells in the spleen and blood of naturally infected dogs and correlated these findings with parasite loads (Silva *et al.* 2014). The results of this study revealed an increase in IL-10 production from Tregs in the spleen of the naturally infected dogs but no correlation could be determined between the percentage of spleen Treg cells producing TGF-*β* or IL-10 and the parasite load (Silva *et al.* 2014). In another study conducted by Cortese *et al*., the immune profiles of dogs naturally infected with *L. infantum* were investigated (Cortese *et al.* 2013). The results revealed a significant increase of Th1 cells, CD8^+^ and CD3^+^ T lymphocytes and a reduced percentage of Treg CD4^+^, CD3^+^ and FoxP3 subsets in infected dogs, irrespective of antibody titre (Cortese *et al.* 2013). Figueiredo *et al*. also looked at naturally infected dogs but reported the expression of Tregs in the jejunum, colon and mesenteric lymph nodes (Figueiredo *et al.* 2014). No correlation was noted between clinical signs and immunological, parasitological findings or histopathological changes in the gastrointestinal tract. However, infection with *L. infantum* resulted in an increased expression of FoxP3, CD4^+^, TGF-*β*, IL-10, TNF-*α* and IFN-*γ* in the colon and jejunum and a reduction of CD8^+^ and IL-4 (Figueiredo *et al.* 2014). Meanwhile, in the skin of naturally infected dogs, Menezes-Souza *et al*. reported a correlation of Tregs GATA-3 and FoxP3 with subclinical infection together with high levels of IL-13, IFN-*γ* and TNF-*α* (Menezes-Souza *et al.* 2011). These findings led the authors to conclude that in subclinical infection or dogs with low levels of skin parasitism, mixed inflammatory and Treg responses could be important for parasite persistence and replication at low levels and also for the maintenance of clinical status of these dogs (Menezes-Souza *et al.* 2011). Hosein *et al*. have also reported the involvement of Tregs, in particular FoxP3 in CanL (Hosein *et al.* 2015). In a recent study, they reported that FoxP3 was positively correlated with parasite density in both the liver and skin of sick dogs in an experimental model of CanL (Hosein *et al.* 2015). Further investigations into the role of Tregs in CanL are still required, but from the information already available it is clear that Tregs are of importance in this infection.

## HUMORAL IMMUNE RESPONSE

In mice, one of the strongest correlations with polarized immunity is the profiling of IgG subclass in antigen-specific immune response (Day, 2007). In humans, associations are dependent on clinical forms of the disease. In localized CL, for example, there was an association with IgG1, IgG2 and IgG3, whereas in diffuse cutaneous forms of the disease the response was dominated by IgG4, IgG1 and IgG2 (Rodriguez *et al.* 1996). Canine leishmaniosis is often associated with a marked humoral response, which is non-protective and denotes failure to control the infection. The levels of *Leishmania*-specific immunoglobulins are greater in sick dogs compared with sub-clinical dogs (Palatnik-de-Sousa *et al.* 2004). A marked association was seen between these levels, tissue parasite density and the clinical status of the animal (Reis *et al.* 2006*b*). Over the years, the levels of canine IgG subclasses; IgG1 and IgG2, have been extensively researched in an attempt to establish a correlation between the type of Th response, the subclass level and the final clinical outcome of infection (Baneth *et al.* 2008). Investigations employing monoclonal antibodies to canine IgG1 and IgG2 have displayed a stable increase in the production of both these subclasses during natural and experimental infection with no indication of a practical use (Quinnell *et al.* 2003*a*; Strauss-Ayali *et al.* 2007). Rodriguez-Cortes *et al*. reported that the intensity of *L. infantum* infection in blood samples was significantly correlated with clinical signs and IgG, IgA and IgM concentrations (Rodriguez-Cortes *et al.* 2007*b*). Understanding humoral responses in CanL dogs is important to establish a good prognosis and to enable the best possible treatment (Papadogiannakis *et al.* 2010).

## INNATE IMMUNE RESPONSES

The components of innate immunity comprise a set of disease-resistance mechanisms that are not pathogen specific but have molecular and cellular components that identify classes of molecules specific to frequently encountered pathogens. Cells associated with innate immunity such as dendritic cells and macrophages directly kill pathogenic microbes via phagocytosis, or they induce the production of cytokines, which facilitate the elimination of pathogens. The innate immune response instructs the development of long lasting pathogen specific adaptive immune responses (Kumar *et al.* 2009).

### Dendritic cells

Dendritic cells are very important in assisting with the control of *Leishmania* infection and impairment of these cells by the parasite often results in the onset of disease. However, limited information is available regarding dendritic cells in canine *L. infantum* infection. Silva *et al*. reported that the impairment of follicular dendritic cells, B cell migration, CXCL13 expression and germinal centre formation were associated with severe clinical forms of CanL (Silva *et al.* 2012). Intradermal injection of leishmanin solution induced antigen-specific maturation of canine dendritic cells in *L. infantum* dogs (Sacchi *et al.* 2006). This had the further effect of inducing up regulation of surface MHC class II expression. The results from this study suggested that canine dendritic cells acted as effector cells in the delayed-type hypersensitivity reaction seen (Sacchi *et al.* 2006).

### Neutrophils

There is now a large amount of evidence, which suggests that *Leishmania* parasites are able to infect and replicate within neutrophils. Polymorphonuclear leukocytes (PMNs) are manipulated so that the parasites use granulocytes as host cells, permitting a productive infection. This enables *L. major* to use the PMNs as ‘Trojan horses’ before entering the macrophages (Laskay *et al.* 2003). Conversely, De Souza Carmo *et al*. showed that amastigote destruction was observed inside *L. amazonensis* infected macrophages that were co-cultured with neutrophils (de Souza Carmo *et al.* 2010). The nitroblue tetrazolium reduction test (NBT) was investigated as a possible quick and cost effective assay for evaluation of percentage of canine neutrophils and monocytes activated in peripheral blood (Gomez-Ochoa *et al.* 2010). The results from the study revealed that dogs with mild disease or no clinical development of disease had a significantly higher neutrophil reactivity (34%) compared with dogs with severe disease (3·7%) (Gomez-Ochoa *et al.* 2010).

Neutrophils have been found to have the ability to de-condense chromatin and eject DNA into the surrounding extracellular environment, resulting in the trapping and inactivation of pathogens (Abi Abdallah and Denkers, 2012). This phenomenon is known as neutrophil extracellular traps (NETs) and recent studies have shown that these NETs are not only important for the destruction of bacterium and viruses but also in protozoan infections including *Leishmania, Eimeria, Plasmodium* and *Toxoplasma* (Baker *et al.* 2008; Guimaraes-Costa *et al.* 2009; Behrendt *et al.* 2010). It has, however, been reported that *Leishmania* parasites have the ability to utilise these NETs to their advantage either by facilitating the containment of the parasite at the site of inoculation (Gabriel *et al.* 2010) or by escaping NET mediated killing by activity of the enzyme 3′-nucleotidase/nuclease (Guimaraes-Costa *et al.* 2014). To date, there are no reports of a role for NETs in CanL.

It has been well established that *L. infantum* infection causes oxidative stress of neutrophils (Almeida *et al.* 2013*b*). Oxidative stress is when there is a disruption in the normal balance between the production of reactive oxygen species and antioxidant defences (Betteridge, 2000). In dogs, oxidative stress was reported to have occurred in both moderate and severe stages of leishmaniosis. The viability of neutrophils decreased in the final stages of the disease and this is thought to have been as a result of uremia (Almeida *et al.* 2013*b*). A similar study also demonstrated that in moderate disease there was increased superoxide production but in very severe stages of disease there was a decrease in superoxide production and increased apoptosis, which was also associated with uremia (Almeida *et al.* 2013*a*).

### Macrophages

Nitric oxide (NO) production is the last step involved in the destruction of *Leishmania* by macrophages. Nitric oxide is produced by nitric oxide synthase (NOS), which converts one of the terminal nitrogens of the guanidine group of L-arginine to NO, producing citrulline (Marletta *et al.* 1988). A number of cytokines have been found to enhance this NO production synergistically with IFN-*α*, potentially facilitating parasite control *in vivo.* IFN-*α* has been found to synergize with IFN-*γ* in the induction of iNOS and NO production by macrophages *in vitro* (Green *et al.* 1990; Rogers *et al.* 2009).

Several studies have demonstrated that the main effector mechanism involved in protection is the activation of macrophages by IFN-*γ* and TNF-*α*, which kill intracellular amastigotes via the L-arginine NO pathway. This has been demonstrated following successful chemotherapy of *L. infantum* dogs (Vouldoukis *et al.* 1996). Anti-leishmanial activity and NO production were also detected in a canine macrophagic cell line infected with *L. infantum* after incubation with IL-2, IFN-*γ* and TNF-*α* (Pinelli *et al.* 2000). Inducible NO synthase (iNOS or NOS2) produces NO and this is known to be one of the main microbicidal mechanisms in murine macrophages. Cells stimulated with human IFN-*γ* and bacterial lipopolysaccharide (LPS) and subsequently infected with *L. infantum* promastigotes showed high levels for fluorescence for NOS2 in their cytoplasm, which was not seen in uninfected macrophages (Sisto *et al.* 2001). The authors concluded that in this infection model, canine macrophages were able to express NOS2 and that IFN-*γ* and LPS have a role in NOS2 production (Sisto *et al.* 2001). Zafra *et al*. investigated the expression of iNOS by *Leishmania* infected canine macrophages and the results from this study revealed that a high iNOS expression was correlated with a low intracellular amastigote burden (Zafra *et al.* 2008). The results from the study suggested that elevated iNOS expression in canine macrophage was a mechanism to control the infection (Zafra *et al.* 2008). Meanwhile, Panaro *et al.* studied dogs naturally infected with *L. infantum* in Southern Italy (Panaro *et al.* 2008). Blood was taken from each dog at 4 month intervals and cell culture experiments were set up using macrophages and lymphocytes obtained from peripheral blood mononuclear cells. Supernatants from cultures were assayed with Griess reagent to detect NO (Panaro *et al.* 2008). The results revealed that in the first few months of infection, NO levels in supernatants with infected macrophages were higher in dogs that were symptomatic compared with asymptomatic dogs. However, 8 months after diagnosis, the level of NO was significantly higher in the asymptomatic group and in addition, the release of NO significantly decreased in cultures with autologous lymphocytes in both groups (Panaro *et al.* 2008). Holzmuller *et al*. demonstrated that the co-incubation of infected macrophages with autologous lymphocytes from dogs, which were immunized with purified excreted-*Leishmania* antigens resulted in cell death of intracellular amastigotes by NO mediated apoptosis (Holzmuller *et al.* 2005). Taken together, these findings re-inforce the notion that NO production is important for parasite elimination.

## TLRs

It has been widely accepted that in susceptible dogs, the innate immune system is evaded by the parasites via a number of mechanisms including remodelling of phagosomal compartments and interference of various signalling pathways (Sacks and Sher, 2002). In particular, within recent years there has been a great deal of interest in the involvement of TLRs in *Leishmania* infection. This has been extensively studied in both human and murine models (Kropf *et al.* 2004*a*; Liese *et al.* 2007; Raman *et al.* 2010; Tuon *et al.* 2010; Gallego *et al.* 2011). However, there is a limited amount of information about the role that these receptors play in canine *L. infantum* infection (Figueiredo *et al.* 2013, 2014; Melo *et al.* 2014*a*). To date, 10 TLRs have been characterized in humans with 12 found to be functional in mice (Kawai and Akira, 2011) and 10 in dogs (Cusco *et al.* 2014). TLRs are a type of pattern recognition receptor. These are type-1 transmembrane proteins, which are expressed by cells of the innate immune system and in turn direct the responses of the adaptive immune system (Venugopal *et al.* 2009). TLRs can induce inflammatory cytokines, type-1 IFN, chemokines and co-stimulatory molecules. The specificity of each TLR is dependent on the cytoplasmic adapter molecules that are associated with the toll- IL 1 receptor (TIR) domain, known as myeloid differentiation primary response gene 88 (MyD88), Mal (MyD88 adapter-like) also known as TIR-containing adapter protein (TIRAP) or toll-like receptor adapter protein (TRAM) (Medzhitov, 2001; Kawai and Akira, 2011). The adaptor molecules in turn provide the necessary structure for the recruitment and activation of downstream kinases and transcription factors that regulate inflammatory responses.

Unlike several other pathogens such as *Listeria* or *Toxoplasma,* there is a marked absence in pro-inflammatory cytokine induction upon phagocytosis of *Leishmania* by macrophages (Carrera *et al.* 1996). Adaptive immunity is indispensable for the resolution of infection. However, there is mounting evidence that it is innate mechanisms, which play an important role in anti-*Leishmania* defences (Faria *et al.* 2012). Early investigations suggesting *Leishmania* induces TLR mediated responses derived from studies using cells lacking MyD88 (Hawn *et al.* 2002). These experiments revealed that *L. major* exposure activated promoter regions of IL-1*α* through the MyD88 pathway in macrophages (Hawn *et al.* 2002). Subsequently it was reported that genetically resistant mice lacking MyD88 had a high susceptibility to *L. major* infection with large non-healing lesions and elevated Th2 responses. These findings suggested TLR responses were important for the development of anti-parasite immunity (Muraille *et al.* 2003).

TLRs have been found to have important roles in several canine diseases such as anal furunculosis (House *et al.* 2008), osteoarthritis (Kuroki *et al.* 2010), inflammatory bowel disease (McMahon *et al.* 2010) and pyometra (Chotimanukul and Sirivaidyapong, 2011) to name a few. Investigations into the importance of TLRs in CanL are ongoing, but at present preliminary. In murine models, TLR2 has been associated with a protective role in leishmaniosis. In a *L. major* murine model, the TLR2 deficient mice had an increased number of cutaneous lesions (de Veer *et al.* 2003). TLR2 and TLR3 were involved in the phagocytosis of *Leishmania donovani* parasites (Flandin *et al.* 2006). In a human neutrophil study, it was reported that TLR2 can directly bind to lipophosphoglycan to promote signalling events (Becker *et al.* 2003). In dogs, it was recently reported that the macrophages derived from peripheral blood from *Leishmania* infected dogs showed a decrease in TLR2 gene expression compared with healthy non-infected dogs (Melo *et al.* 2014*b*). However, Amorim *et al.* described high TLR2 expression in monocytes and granulocytes in *Leishmania* infected dogs in Brazil. These dogs were not infectious to sand-flies by xenodiagnosis as the skin *Leishmania* parasite burden was found to be insufficient (Amorim *et al.* 2011). More recently, TLRs in CanL were studied in the gastrointestinal tract of infected dogs. This study revealed that the immunological and parasitological parameters varied by section of the gastrointestinal tract (Figueiredo *et al.* 2013). There was a high parasite load in the colon, as well as higher frequency and expression of TLR2, CD11c receptors and IL-4 (Figueiredo *et al.* 2013). A study by Ordeix *et al*. recently revealed that there was a lower expression of TLR2 in skin biopsies from dogs with mild disease (papular dermatitis) compared with dogs with moderate or severe disease, supporting an association between TLR2 and the pathogenesis of cutaneous lesions in CanL (Ordeix *et al.* 2015). Hosein *et al.* reported down regulation of TLR2 transcription in the early stages of infection in lymph node samples of dogs experimentally infected with *L. infantum*, followed by up regulation in subclinical but not sick dogs. Further evidence of up regulation of TLR2 was revealed in the liver of both infected groups when compared with non-infected controls (Hosein *et al.* 2015). Overall, TLR2 up regulation seems to be associated with disease progression in dogs.

TLR3 is reported to be central in the production of NO and parasite phagocytosis (Flandin *et al.* 2006). *In vitro* studies have implied that there is a possible role for TLR3 in immunity against *Leishmania* (Flandin *et al.* 2006) but *in vivo* data is scarce, even in the mouse model. It is believed that a down regulation in this TLR would favour disease progression. In a hamster model, Ives *et al*. demonstrated that metastasizing *L. guyanensis* parasites have a high *Leishmania* RNA virus–1(LRV1) burden, which is recognized by the host TLR3 to induce pro-inflammatory cytokines and chemokines and exacerbate disease (Ives *et al.* 2011). There is even less information regarding TLR3 in CanL. A recent study by Turchetti *et al*. looked at the transcription of cytokines (IL-10, IL-12, TNF-*α* and IFN-*γ*) and innate immunity genes (Nramp1, Nod-like receptor (NOD)1, NOD2, TLR1, TLR2, TLR3, TLR4, TLR5, TLR6, TLR7 and TLR9) in canine macrophages that were resistant or susceptible to intracellular survival of *L infantum.* The study revealed that the decreased intracellular survival of *L. infantum* in macrophages was associated with increased production of IFN-*γ* and TNF-*α*, and decreased production of IL-10 (Turchetti *et al.* 2015). This supports the notion that resistance is associated with a Th1-type response (Pinelli *et al.* 1994). However, TLR, NLR or Nramp1 appeared to have no influence on the intracellular survival of *L. infantum* (Turchetti *et al.* 2015). In a recent experimental CanL study, it was reported that TLR3 transcription was significantly downregulated with progression of disease in the lymph nodes when compared with control non-infected dogs and a tendency of down regulation was also noted in skin and spleen tissues (Hosein *et al.* 2015). As the information relating to TLR3 in CanL is so scarce, further studies would be required to be able to ascertain if this TLR has an important role in this infection.

TLR4 has been described as having a protective role in *L. major* infections. TLR4 deficient mice had a diminished parasite load in skin lesions but an increased parasite survival in host cells, which was correlated with higher arginase activity (Kropf *et al.* 2004*a*). The same group later showed that there was an increased parasite growth and delayed healing of cutaneous lesions resulting from a lack of TLR4 (Kropf *et al.* 2004*b*). In a *L. infantum* mouse model study, it was revealed that *L. infantum* infection resulted in increased transcription of TLR4 along with TLR2 (Cezario *et al.* 2011). This increased transcription could have been due to an influx of inflammatory cells into the spleen, in particular at the beginning of the infection, and a decrease seen at the chronic stage of infection could possibly have been due to partial control of the infection (Cezario *et al.* 2011). In a recent study looking at naturally infected dogs, Melo *et al.* reported increased TLR2 and TLR4 expression in the spleens of naturally infected dogs compared with non-infected dogs (Melo *et al.* 2014*a*). This elevated expression could indicate that these TLRs are important for the control of the infection and they are elevated in an attempt to eliminate the parasite. More recently, it was reported that significant changes were seen with TLR4 transcription in both the lymph node and spleen in an experimental model of CanL (Hosein *et al.* 2015). In both these tissues, TLR4 was significantly downregulated in the early (subclinical infection) as well as the late stages of infection (disease progression) when compared with non-infected controls (Hosein *et al.* 2015). TLR-4 down regulation appears to be associated with disease progression in dogs.

In a *L. infantum* murine model, cytokine production from dendritic cells was dependent on TLR9 (Liese *et al.* 2007) and in *L. major* infection, TLR9 was essential for natural killer cell response (Liese *et al.* 2007). Down regulation of the TLRs with disease progression is suggestive of an inhibitory role where the parasite may be facilitating the onset of disease by reducing or limiting the transcription of these TLRs, which would otherwise play a protective role.

The expression of TLR1, TLR3, TLR4 and TLR7 were assessed in both TLR2 and TLR9 deficient macrophages (Pandey *et al.* 2015). The results of the study revealed that TLR9 was important for the modulation of TLRs 1, 2 and 3 in a *L. major* macrophage model. The TLR9 deficient macrophages in this study also had reduced CD40 expression and lower levels of TNF-*α* and IL-12 (Pandey *et al.* 2015). Tuon *et al*. looked at TLR9 expression in patients with cutaneous leishmaniosis caused by *L. braziliensis* and found that TLR9 expression was mainly observed in granulomas. Some TLR9 positive cells were found in the dermis of patients. The percentage of cells that expressed TLR9 was significantly higher in the skin of CL patients compared with normal skin, but TLR9 expression was downregulated in keratinocytes of infected patients (Tuon *et al.* 2008). In contrast, Hosein *et al.* reported significant changes in TLR9 transcription in the spleen and skin of experimentally infected dogs. TLR9 transcription was only upregulated in the early stages of infection in both these tissues when compared with healthy non-infected control dogs (Hosein *et al.* 2015), while TLR9 down regulation was observed in lymph nodes and skin with disease progression (Hosein *et al.* 2015). In another canine study, increased frequency and expression of TLR9 was associated with a lower parasite load in the jejunum of *L. infantum* infected dogs, whereas the colon showed a higher parasite load along with an increased frequency and expression of TLR2 (Figueiredo *et al.* 2014). Therefore, in CanL, TLR9 down regulation appears to correlate with disease progression. Table 2 summarizes the work to date on TLRs in CanL.

**Table 2.** Summary of published studies evaluating TLRs in canine *L. infantum* infection

TLRs/tissues evaluated	Technique employed for TLR detection	Clinical status classification and number of dogs studied	Geographical location	Main findings	Reference
TLR2, TLR3, TLR4, TLR9/lymph node, liver, spleen, skin	RT–PCR	Healthy non-infected (*n* = 10)	Spain and UK	↓TLR3, TLR4 and TLR9 within lymph nodes with disease progression	(Hosein *et al.* 2015)^a^
		Sub-clinical (*n* = 24)		↓TLR4 in the spleen in infected dogs	
		Sick (*n* = 7)		↑TLR9 in the skin in early stages of infection	
				↑TLR2 with disease progression in liver and lymph nodes	
TLR2/skin	Immunohistochemistry	Healthy non-infected (*n* = 6)	UK and Spain	↓TLR2 was revealed in skin biopsies from dogs with stage I compared with dogs with stages II and III	(Ordeix *et al.* 2015)^b^
		Stage I, mild disease (*n* = 11)			
		Stage II and III, moderate and severe disease (*n* = 10)			
TLR2, TLR4/PBMC	Flow cytometry	Healthy controls (*n* = 60)	Brazil	↓ TLR4 in SD	(Melo *et al.* 2014*b*)^b^
		SD (*n* = 60)		No changes observed with TLR2	
TLR2, TLR4, TLR9/brain, choroid plexus, spleen, lymph node	RT–PCR	Healthy non-infected (*n* = 4) SD with no neurological signs (*n* = 15)	Brazil	↑ TLR2 and TLR9 in the choroid plexus in infected dogs, whereas this was only the case for TLR2 in the brain	(Melo *et al.* 2014*a*)^b^
				↑ TLR2 and TLR4 in infected dogs in both spleen and lymph node	
TLR2, TLR9/jejunum, colon	Flow cytometry	Non-infected dogs (*n* = 6)	Brazil	↑TLR2 and parasite load in the colon compared with the jejunum	(Figueiredo *et al.* 2013)^b^
		AD (*n* = 12)			
		SD (*n* = 12)		↑ TLR9 in the jejunum than the colon	
TLR2/blood	Flow cytometry	SD (*n* = 48)	Brazil	↑ Nitric oxide and, CD11^b+^ and TLR2 in dogs with Xeno-/IHQ-	(Amorim *et al.* 2011)^b^
		Xeno+/IHQ+ (*n* = 17)			
		Xeno-/IHQ− (*n* = 16)			

TLR, toll-like receptor; RT–PCR, reverse transcriptase and real time polymerase chain reaction; PBMC, peripheral blood mononuclear cells; Xeno, xenodiagnosis; IHQ, immunohistochemistry; AD, asymptomatic; SD, symptomatic.

a Natural infection.

b Experimental infection.

It is evident that there is a very important role for TLRs in protection against *Leishmania* infections including *L. infantum* infection in dogs. So far, there is a strong association between overall TLR down regulation and disease progression, with the exception of TLR2 in dogs. These parasites have developed strategies to hijack the innate immune responses in a survival attempt.

Moreover, several research groups have employed the strategy of directly targeting TLRs using specific agonists to elicit protection against several species of *Leishmania* including *L. major* and *L. donovani*. In particular, this has been trialled and success reported for TLRs 2, 4 and 9. Stimulation of these TLRs elicited a protective response and it has been suggested that antigens stimulating TLRs would be ideal as potential vaccines (Chandra and Naik, 2008; Raman *et al.* 2010; Srivastava *et al.* 2013; Chandel *et al.* 2014; Huang *et al.* 2015). In *Leishmania,* there has already been some progress with the use of ligands in vaccine development using mice models, which is promising for future dog studies. Most recently, Craft *et al.* reported the use of topical resiquimod (a synthetic TLR7/8 activating molecule), inducing protection against *L. infantum* infection in mice (Craft *et al.* 2014). Topical application to the skin of mice prior to, or following systemic infection resulted in conferred resistance to future intravenous challenge and protection that persisted for as long as 8 weeks after the first topical treatment (Craft *et al.* 2014). Mice with existing infections were also found to have significantly lower visceral parasite loads following topical resiquimod treatment. Resiquimod was found to increase the trafficking of leukocytes, B-cells, dendritic cells, macrophages, granulocytes, CD4^+^ and CD8^+^ T cells in livers and spleens of the mice (Craft *et al.* 2014). In an earlier study, subcutaneous vaccination with imiquimod, a TLR7/8 agonist, was shown to mediate a Th1 response against *L. major* antigen, which suppressed Th2 responses following a challenge infection (Zhang and Matlashewski, 2008). Both studies suggested the use of these compounds as potential vaccine adjuvants for vaccines or topical preventative and therapeutic agents (Zhang and Matlashewski, 2008; Craft *et al.* 2014).

## IMMUNOMODULATION: FROM PREVENTION TO TREATMENT

Effective control of CanL needs to address the vertebrate host, the vector and the parasite. Life cycle interruption of *Leishmania* is currently best achieved through the use of permethrin based impregnated dog collars or the topical application of insecticides (Killick-Kendrick *et al.* 1997; Courtenay *et al.* 2009). Spot-on treatment of permethrin was also found to significantly reduce the incidence of CanL (Manzillo *et al.* 2006*a*). Additional control measures include spraying homes, animal shelters and soft furnishings with pyrethroids (Alexander and Maroli, 2003). Ultimately, the best control strategy would be an effective vaccine. Such a vaccine would not only reduce the number of CanL cases but also the incidence in humans (Alvar *et al.* 2004). Several vaccine candidates have been tested in the past couple of decades with varying rates of success in dogs (Foroughi-Parvar and Hatam, 2014). There is no clear cut immune response in CanL, but it is well established that a Th1 dominated response is the most desired outcome for protection in the dog (Baneth *et al.* 2008).

Currently, in Brazil, there is one commercially available canine vaccine; Leish-Tec^®^ (Hertape Calier Saude Animal SA). There were previously two, but Leishmune^®^ (Fort Dodge Animal Health) is no longer available. Leish-Tec^®^ appeared to partially protect dogs against CanL, but only limited data from field trials have been reported (Fernandes *et al.* 2008). The same authors later reported that dogs vaccinated with Leish-Tec^®^ exhibited adverse reactions with greater frequency and severity compared with dogs vaccinated with Leishmune^®^ (Fernandes *et al.* 2014). The toxic effects of saponin are believed to vary depending on the compound purity and the dosage used. Both these vaccines contained saponin as an adjuvant, but Leishmune^®^ was formulated with purified fraction QS21 (Oliveira-Freitas *et al.* 2006), known to have milder toxic effects (Kensil *et al.* 1991). In earlier trials, Leishmune^®^ was found to induce a significant, long-lasting protective effect against CanL in Phase III clinical trials (da Silva *et al.* 2000; Borja-Cabrera *et al.* 2002). Leishmune^®^ was also proposed to be used as a transmission blocking vaccine and in immune therapy for dogs already infected (Borja-Cabrera *et al.* 2004; Saraiva *et al.* 2006).

In Europe, the LiESP/QA-21 vaccine was recently made available. This vaccine is marketed under the trade name CaniLeish^®^ (Virbac, France) and is composed of purified excreted-secreted proteins of *L. infantum* (LiESP) and adjuvanted with QA-21, which is a purified fraction of the *Quillaja saponaria* saponin (Lemesre *et al.* 2007). CaniLeish^®^ is licensed for use with a primary vaccination course comprising three injections at 3-week intervals subsequently followed by annual booster vaccinations (Moreno *et al.* 2012). The same authors were able to demonstrate *in vitro* that CaniLeish^®^ elicited a protective Th1 dominated immune profile, which lasted for a year after vaccination, and macrophages were able to reduce the intracellular parasite burdens in cell culture assays when co-cultured with autologous lymphocytes (Moreno *et al.* 2014).

Another study evaluated the preventative efficacy of a domperidone based treatment programme against CanL in a high prevalence area (Sabate *et al.* 2014). Domperidone is a dopamine D2 receptor antagonist that has been shown to enhance the innate CMI response, mainly through the reversible increase of blood prolactin (Gomez-Ochoa *et al.* 2009). The results from the study demonstrated that this treatment regime reduced seroconversion rates in treated healthy dogs in endemic areas of CanL (Sabate *et al.* 2014).

## TREATMENT OF CLINICAL DISEASE

There are several drugs currently available for the treatment of CanL. However, while many of these drugs can result in improved clinical signs, none of them have the ability to eliminate the infection. There is also the issue of toxicity and drug resistance (Noli and Auxilia, 2005). The most common drugs used for CanL are the meglumine antimoniate, miltefosine and allopurinol. However, in recent years attempts have been made to use immumodulatory molecules for treatment of CanL such as the protein aggregate magnesium-ammonium phospholinoleate-palmitoleate anhydride immumo-modulator (P-MAPA) (Santiago *et al.* 2013) that appears to induce TLR2 in human embryonic kidney (HEK) cells (Favaro *et al.* 2012) and in canine macrophages (Melo *et al.* 2014*b*). The treatment with this immumodulator resulted in an improvement in clinical signs and a significant reduction in parasite load in the skin. Peripheral blood mononuclear cells (PBMC) cultures also showed an increase in IL-2 and IFN-*γ* and a decrease in IL-10 levels (Santiago *et al.* 2013). Taken together, the findings from this study indicated that P-MAPA has the potential to be used as an immunotherapeutic drug in CanL (Santiago *et al.* 2013). Another study reported the use of a recombinant cysteine proteinase from *L. infantum* (rLdccys1) for immunotherapy of CanL (Ferreira *et al.* 2014). Dogs treated with this proteinase did not display worsening clinical signs, had a reduction in the splenic parasite load, high IFN-*γ* and low IL-10 concentrations in comparison with control dogs (Ferreira *et al.* 2014). The findings of this study illustrated the potential use of rLdccys1 as an immunotherapeutic tool for CanL and warrants further investigations (Ferreira *et al.* 2014).

In conclusion, understanding of CanL caused by *L. infantum* is complicated. Visceral dissemination of the parasites makes sampling from infected animals difficult. In response experimental *in vivo* studies using mice have commonly been undertaken, although unfortunately these offer a poor reflection of immune responses associated with *L. infantum* in dogs. It is essential that as much information as possible be gathered from both naturally and experimentally infected dogs to better our understanding of this disease. Taken together, the findings summarized in this paper illustrate the multi-systemic effect of *L. infantum* in dogs. Although a great deal of progress has been made towards understanding the immune responses associated with CanL, a vast amount remains to be determined. A more comprehensive understanding of both the local and systemic responses to *L. infantum* in the dog is needed to be able to develop more effective treatment regimens and/or vaccines. Improving the control of the spread of CanL might also have a positive impact on reducing the number of human cases of the disease.
